# Evaluating the Biological Properties of Chitosan and Gelatin-Based Scaffold in Saliva and Blood: An In Vitro Study

**DOI:** 10.7759/cureus.48667

**Published:** 2023-11-11

**Authors:** Vyshnavi B Sindhusha, Jayakumar N Doraiswamy

**Affiliations:** 1 Periodontics, Saveetha Dental College and Hospitals, Saveetha Institute of Medical and Technical Sciences, Chennai, IND

**Keywords:** anticoagulant-added blood, saliva, scaffold, gelatin, chitosan

## Abstract

Aim

Guided tissue regeneration (GTR) membranes have been used to encourage the regrowth of periodontal tissues, but traditional GTR membranes may not degrade at the right rate, which can lead to complications. Multifunctional scaffolds such as chitosan and gelatin-based scaffolds are designed to have controlled biodegradation, ensuring that they remain in place long enough to support tissue regeneration. These scaffolds are included with hemostatic properties that can help control bleeding, making the procedure safer for patients. Hence, the aim of the study was to evaluate the biological properties such as biodegradability, adsorption, and surface characteristics of chitosan and gelatin-based scaffolds immersed in different body fluids using scanning electron microscopy (SEM) analysis.

Materials and methods

The biodegradability of the chitosan and gelatin-based scaffold was seen along with the commercially available PerioCol®-GTR (Eucare Pharmaceuticals Private Limited, Thirumudivakkam, Tamil Nadu, India), fish collagen. The scaffold was cut into equal sizes and immersed in different body fluids. Then the samples were incubated at 37 degrees for 48 hours and subjected to SEM analysis to assess the surface characteristics and the adsorption of the body fluids onto the surface of the scaffold.

Results

The biodegradability was less than a 7% decrease in weight in the test group (chitosan and gelatin-based scaffold) and a 13% decrease in weight in the control group (PerioCol). The SEM analysis showed that the scaffold had smooth surface morphology and porous nature along with greater adsorption potential (175.8±0.05, 52.02±0.01, and 425.4±0.3 micrometers) in various body fluids.

Conclusion

Chitosan and gelatin-based scaffolds have slower biodegradability when compared with the PerioCol. The scaffold shows good surface morphology and higher adsorption when immersed in various body fluids.

## Introduction

Periodontitis is characterized by chronic inflammation of the periodontal tissues and it involves cycles of inflammation in response to bacterial infection followed by periods of immune suppression of the infection. Progress in periodontitis often leads to the destruction of the supporting structures around teeth such as bone and connective tissues [1]. The primary goals of periodontal treatments include reducing harmful bacteria and inflammation along with control of disease progression and prevention of recurrence. There were different approaches to periodontal treatment which encompassed both non-surgical techniques, surgical techniques, and regenerative procedures [2]. 

Guided tissue regeneration (GTR) is a procedure that utilizes a specialized membrane to facilitate the regeneration of lost periodontal tissue [3]. The primary goal of the GTR membrane is to selectively guide cellular proliferation. The membrane serves as a barrier to prevent certain cell types such as epithelial cells into the wound area, thus encouraging the growth and migration of cells that are essential for tissue regeneration [4]. The average pore size of the membrane should be greater than 100 micrometers with a porosity range>70% to improve the transport of nutrients and bone growth [5]. GTR membranes can be made from natural or synthetic polymers. The variation in the degradability of membranes led the researchers to newer natural polymers such as chitosan and gelatin [6]. Chitin is a naturally occurring amino polysaccharide composed of glucosamine and N-acetylglucosamine seen in the exoskeletons of crustaceans and insects. The biomaterial named chitosan is derived from chitin through partial deacetylation [7]. It is biocompatible and biodegradable in nature, exhibits antimicrobial properties, and is useful in various biomedical applications such as tissue engineering and regenerative medicine; it is also known for minimal host immune rejection, which makes it useful in medical devices and implants [8]. The material can form cationic clusters, which can be beneficial for nerve regeneration, bone repair, and wound healing [9]. This biomaterial acts as a substrate that resembles glycosaminoglycans found in the bone extracellular matrix and thus helps in promoting bone tissue growth and regeneration [10].

Gelatin is considered as a natural biopolymer which is derived from collagen through hydrolysis. Gelatin is known for its excellent biocompatibility and biodegradability [11]. It is non-antigenic and non-toxic, making it safe for use in biomedical applications. Gelatin is described as adhesive and promotes the adhesion and migration of bone cells to the scaffold. The unique sequence of glycine, proline, and hydroxyproline, functions as a contributing factor in the ability of gelatin to promote bone cell adhesion and migration [12]. These amino acids may enhance cell interactions with the material. Gelatin is blended with chitosan to create composite scaffolds and improve the biological activity of the scaffold, making it suitable for tissue engineering and regenerative medicine applications [13]. 

In this study, the biodegradability, adsorption, and surface characteristics of chitosan and gelatin-based scaffolds in different body fluids were assessed. The biodegradability of the scaffold was evaluated against PerioCol®-GTR (Eucare Pharmaceuticals Private Limited, Thirumudivakkam, Tamil Nadu, India), fish collagen, and the adsorption of the scaffold was assessed by studying the surface characteristics of the scaffold using scanning electron microscopy (SEM) analysis.

## Materials and methods

The present study was conducted at the research lab for material sciences located in Saveetha Dental College and Hospitals, Chennai, India, after obtaining approval from the Scientific Review Board of Saveetha Dental College and Hospitals (SRB/SDC/PERIO-2104/23/091). The study includes the test for the biodegradability and surface characteristic evaluation of the chitosan and gelatin-based scaffold. The biodegradability test was done by noting the changes in weight between the test and control groups. Chitosan and gelatin-based scaffold and PerioCol were used as test and control samples. The surface characterization was done by immersing the chitosan and gelatin-based scaffold in different body fluids and studying the surface characteristics of the scaffold with the help of SEM images.

Methodology

Biodegradability

**Figure 1:**
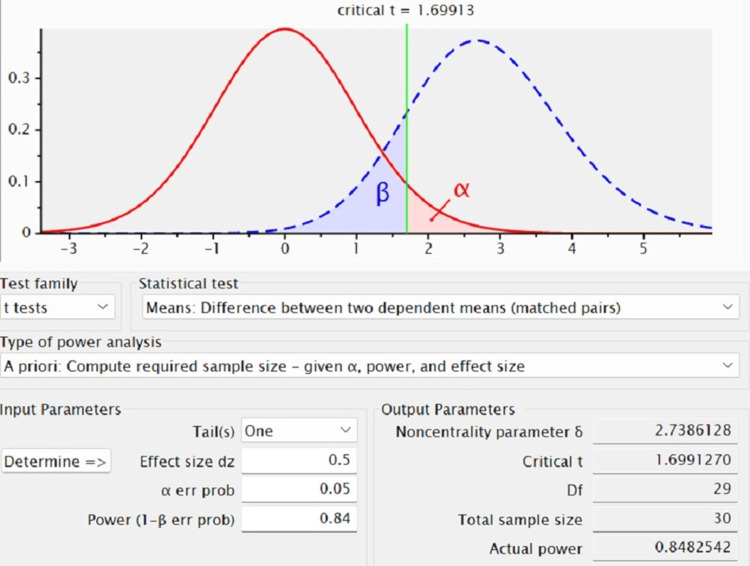
Sample size calculation using G*Power software G*Power software (Heinrich Heine University Düsseldorf, Düsseldorf, Germany) calculation was done to obtain the sample size to check the biodegradability of the scaffold against the membrane.

The biodegradability of the scaffold was evaluated against the membrane by immersing the materials in artificial saliva as it is the sterile solution that is used to study the biodegradation of metal alloys and pharmaceutical drugs in medical and dental fields. A total of 30 samples were taken after the sample size calculation using G*Power software (Heinrich Heine University Düsseldorf, Düsseldorf, Germany) (Figure 1); these were equally segregated into test (chitosan and gelatin-based scaffold) and control (PerioCol) groups.

The samples were individually weighed, with each group having an initial weight of approximately 45 milligrams. Then these samples were immersed in 20 ml of artificial saliva and incubated at 37 degrees centigrade over a time period of four weeks (28 days). During this period, the samples were tested for their weights at specific intervals: one day, seven days, 14 days, and 28 days. 

Characteristics of Chitosan and Gelatin-Based Scaffold

**Figure 2:**
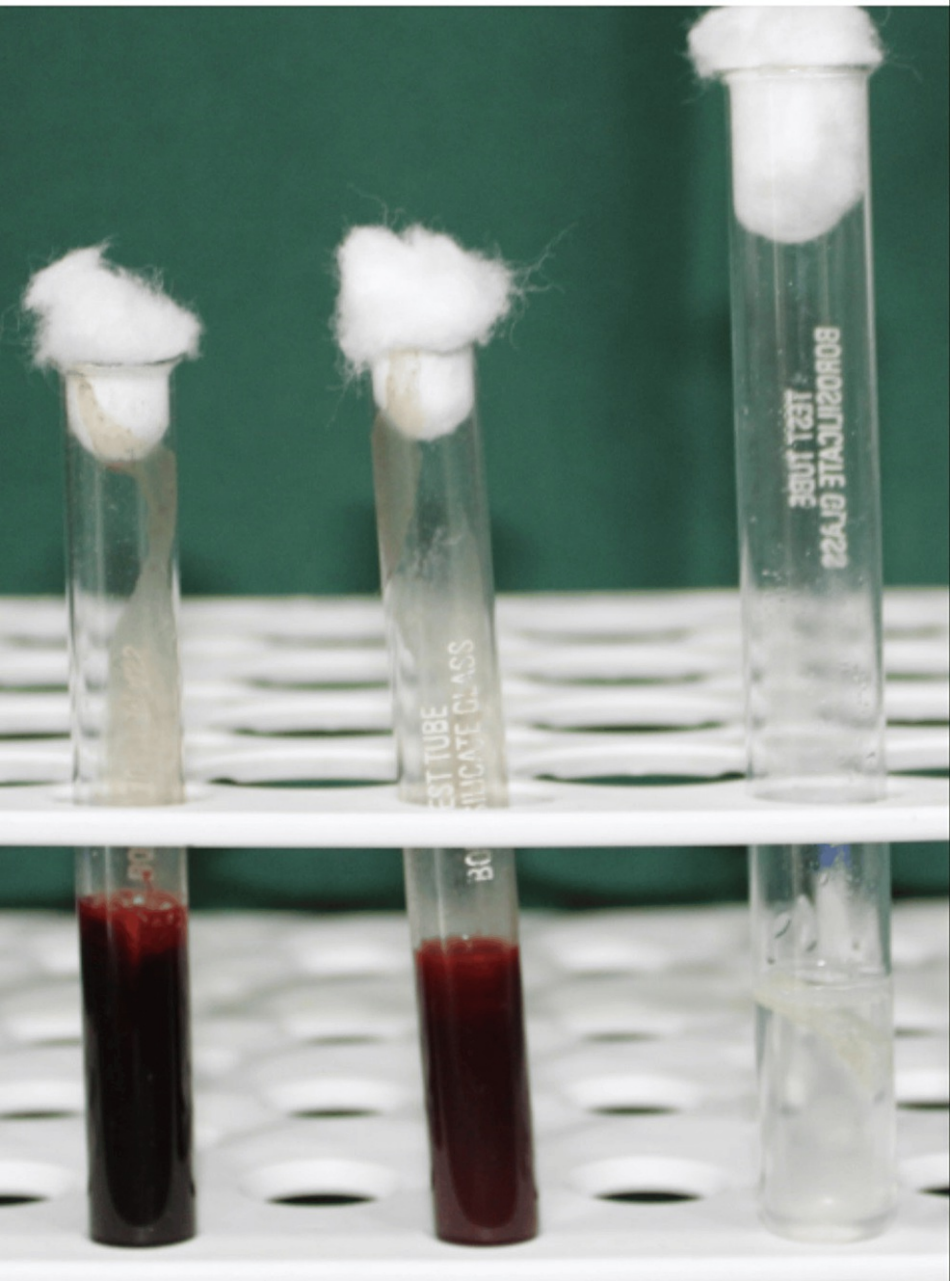
Chitosan and gelatin-based scaffold immersed in various body fluids such as saliva, blood, and anticoagulant-added blood

The body fluids were harvested from 30 healthy individuals (sample size) after obtaining informed consent from the participants. Chitosan and gelatin-based scaffold was cut into equal sizes of 20mm and immersion in various body fluids, saliva, blood, and anticoagulant-added blood, was done for a time period of 48 hours (Figure 2) and then incubation at 37 degrees centigrade was performed.

After the 48-hour immersion period, the membranes were subjected to freeze-drying using a lyophilizer. After lyophilization, the scaffold samples were cut into equal sizes of 20mm each and used for SEM analysis with Hitachi S-4800 scanning electron microscope (Hitachi, Ltd., Chiyoda City, Tokyo, Japan), where the surface morphology of samples was studied. The microscope was operated at a voltage of 3.5 kilovolts. The prepared chitosan and gelatin-based scaffold samples were placed onto a test stage within the microscope for observation; the total acquisition time for each sample was 15 minutes when SEM images were obtained. 

## Results

Biodegradability test

**Figure 3:**
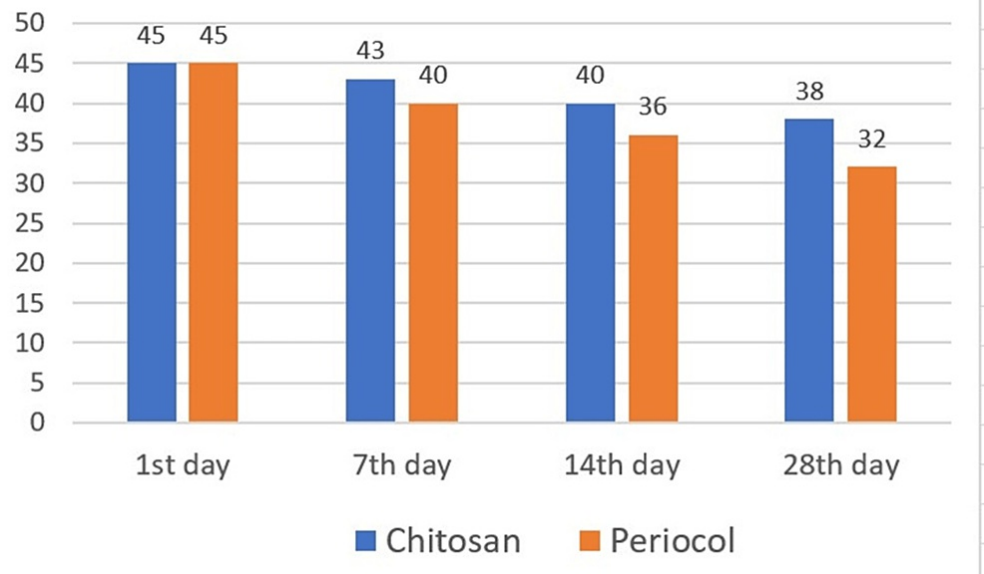
Biodegradability of the test and control group membranes over a period of four weeks

The biodegradability of the scaffold was evaluated in an in vitro setting where a total of 30 samples of the membranes (15-test group, 15-control group) were immersed in artificial saliva. The changes in the weight of the membrane between the test and control groups were measured by weighing the membranes during the first, seventh, 14th, and 28th days. The major variation in the weight of the control group membrane was seen by the seventh day and the basic structure of the PerioCol became more friable as the time progressed. The test group membrane showed a major variation in the weight by the 14th day followed by an accelerated reduction in the weight of the test membrane (Figure 3).

The mean value along with the standard deviation of the biodegradability was given, where a paired t-test was used to measure the statistical difference between groups, and a p-value of<0.05 was seen, which was considered to be statistically significant (Table 1).

**Table 1:** Biodegradability of the test membrane along with its porosity percentage *PerioCol®-GTR (Eucare Pharmaceuticals Private Limited, Thirumudivakkam, Tamil Nadu, India)

Immersion time period (number of days)	Test group (chitosan and gelatin-based scaffold) in milligrams	Loss of membrane weight in percentage (chitosan and gelatin-based scaffold)	Control group (PerioCol) in milligrams	Loss of membrane weight in percentage (PerioCol)
1^st^ day	45.8±0.9	0.4%	45.3±0.3	0.5%
7^th^ day	43.2±1.3	2%	40.2±1.6	5%
14^th^ day	40.8±0.6	5%	36.4±0.5	9%
28^th^ day	38.6±2.1	7%	32.1±1.8	13%

Until four weeks, the chitosan and gelatin-based scaffold maintained its basic architecture. On the other hand, the PerioCol membrane showed a faster rate of degradability, losing 13% of its original weight, whereas the chitosan and gelatin-based scaffold lost less than 7% of its original weight. The result states that the degradation rate of chitosan and gelatin-based scaffold was way slower than the commercially available PerioCol. 

Surface characteristics of chitosan and gelatin scaffold

**Figure 4:**
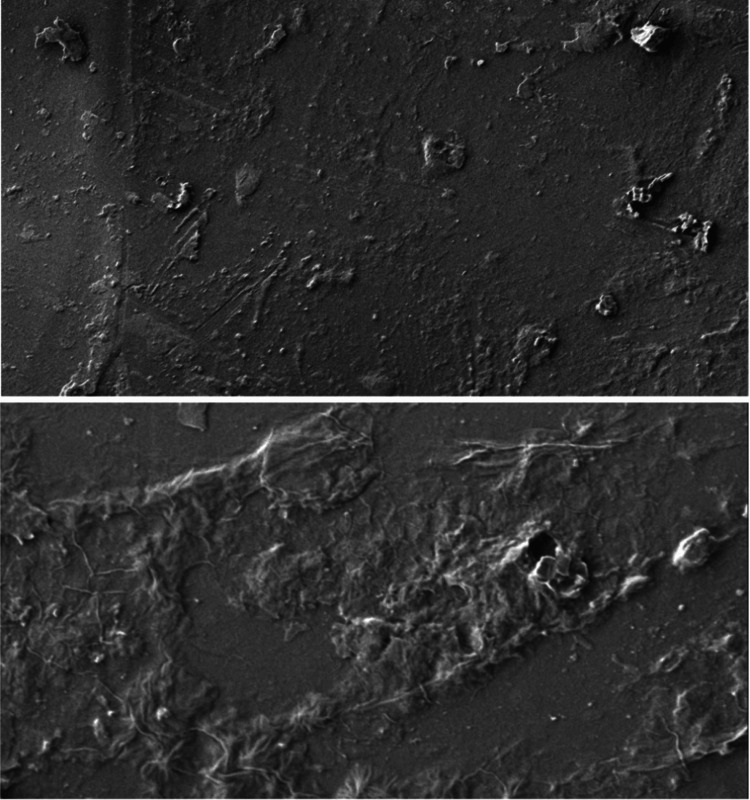
Surface characteristics of the scaffold in saliva This scanning electron microscopy (SEM) image with a resolution of 1250nm shows a translucent surface with an even distribution of salivary proteins onto the scaffold.

**Figure 5:**
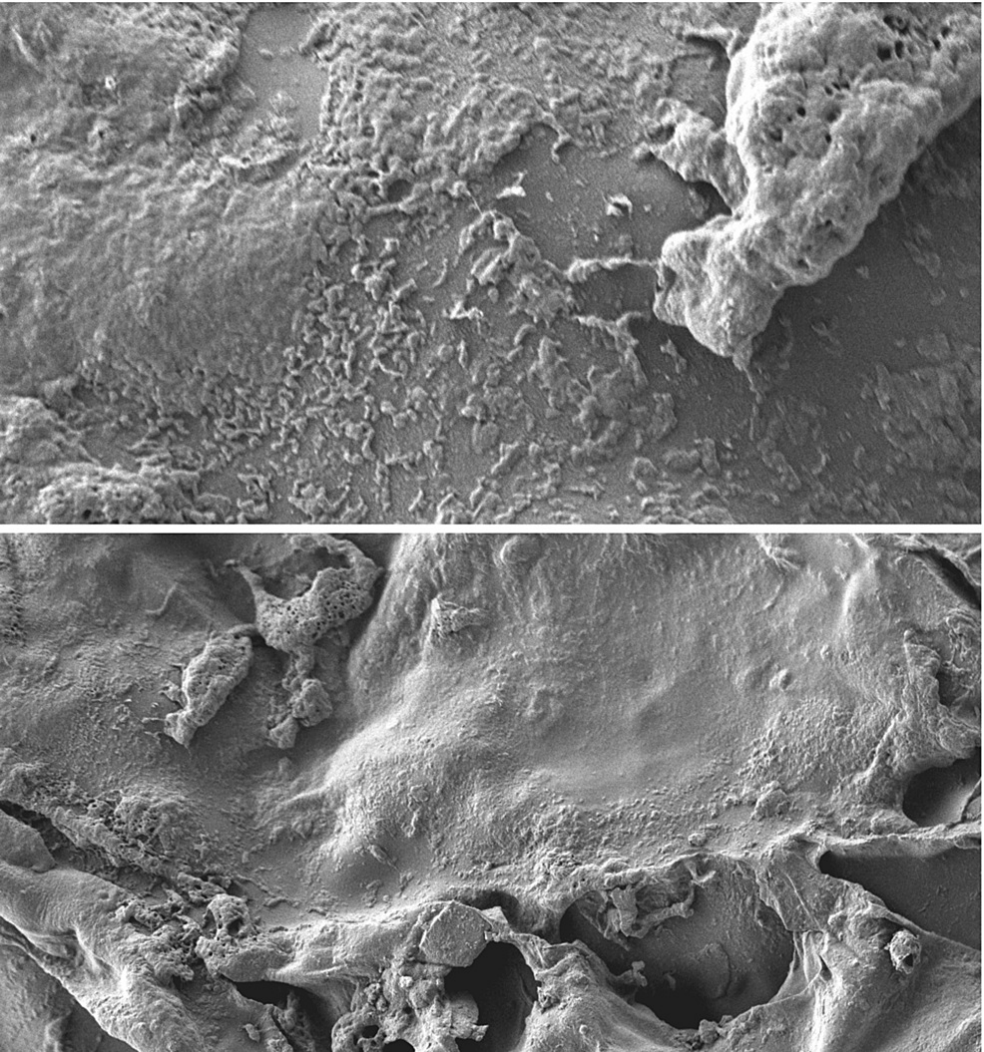
Surface characteristics of the scaffold in blood This scanning electron microscopy (SEM) image with a resolution of 1250nm shows a rough and irregular surface with uneven coagulated proteins of blood onto the scaffold.

**Figure 6:**
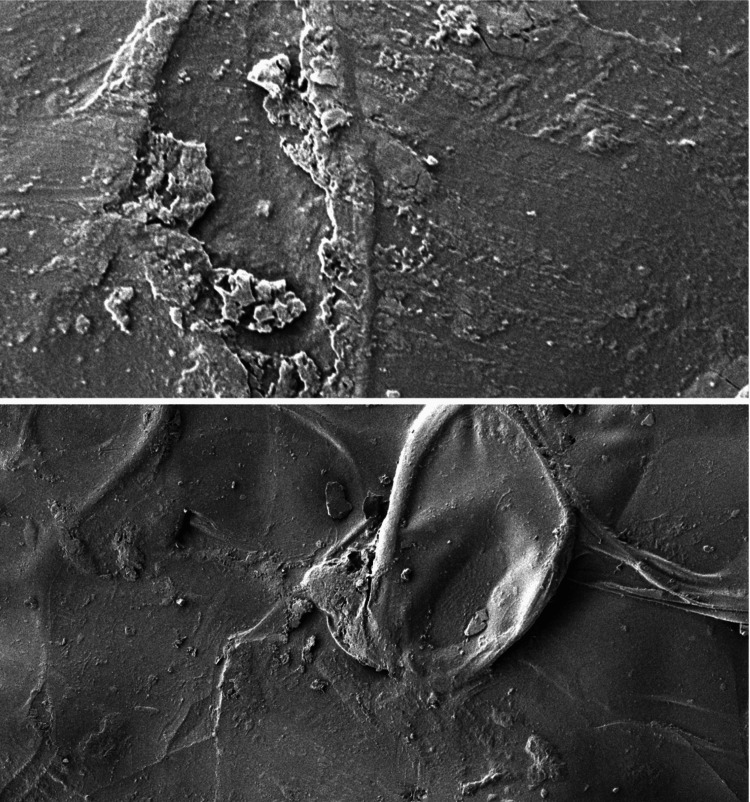
Surface characteristics of the scaffold in anticoagulant-added blood This scanning electron microscopy (SEM) image with a resolution of 1250nm shows an even surface with an equal distribution of coagulated proteins onto the scaffold.

The scanning electron microscope images show the surface characteristics of the chitosan and gelatin-based scaffold immersed in different body fluids such as saliva (Figure 4), blood (Figure 5), and anticoagulant-added blood (Figure 6).

When immersed in saliva, the scaffold had a smooth and translucent surface with an even distribution of salivary proteins onto the surface of the scaffold. In anticoagulant-added blood, the surface of the scaffold was coated with various haematologic proteins and coagulation factors, which showed an even distribution on the surface. In the case of blood, a rough and irregular surface was seen with an uneven distribution of the coagulated proteins. The result of the SEM images states that the surface of the scaffold has better adsorption to various body fluids such as saliva, blood, and anticoagulant-added blood.

The porous structure and the adsorption of the fluids onto the surface of the scaffold are seen in Table 2.

**Table 2:** Pore size of scaffold in various body fluids

Different body fluids	Pore size (mean±SD) in micrometers	Porosity in percentage	P-value
Saliva	175.8±0.05	85%	0.03
Blood	52.02±0.01	72%	0.02
Anticoagulant-added blood	425.4±0.3	80%	0.05

The mean pore size of the chitosan and gelatin-based scaffolds was around 175.8±0.05 micrometers with mean porosity estimated at around 85% in saliva. The scaffold showed a suitable porous structure with a pore size of 425.4±0.3 micrometers and 80% porosity when immersed in anticoagulant-added blood. The scaffold immersed in blood showed a mean pore size of 52.02±0.01 micrometers and 70% porosity. The comparison between the groups was done using Student’s t-test with the p-value<0.05, which is considered statistically highly significant.

## Discussion

Barrier membranes play a crucial role in the regeneration of periodontal tissues and the facilitation of bone augmentation, particularly in the context of implant treatments. The use of barrier membranes is supported by research and clinical protocols like GTR and guided bone regeneration (GBR) [14]. The first demonstration of the formation of new attachment to teeth in humans through periodontal tissue regeneration using barrier membranes was given by Nyman et al. in 1982 [15]. The further progress in the barrier membranes has led to their use in specific operation protocols, such as GTR and GBR. Barrier membranes used for tissue regeneration can be categorized based on the biodegradability of their base materials [16].

Chitosan and gelatin are versatile biomaterials with numerous applications in the field of tissue engineering. These biomaterials are used in creating scaffolds that provide a three-dimensional structure that mimics the natural extracellular matrix of tissue, supporting the growth and regeneration of periodontal ligament (PDL) cells [17]. They are noted for their ability to form a polyelectrolyte complex, which has implications for cell interactions and tissue regeneration processes within the scaffold. The pore size of the chitosan and gelatin-based scaffold was greater than 250 micrometers and this pore size helps in the adsorption of the proteins and transportation of the fluids [18]. Both chitosan and gelatin are biocompatible and biodegradable, and have the ability to promote cell adhesion and migration facilitating tissue growth and repair [19]. 

In this study, the biodegradability test of the chitosan and gelatin was measured against PerioCol. The scaffolds were immersed in artificial saliva for a time period of four weeks and the weight of the scaffold was measured at a time interval of seven days; at the end of four weeks, the difference in the proportions of the weight between the chitosan and gelatin scaffold against the PerioCol was highly significant with 8% of weight loss between the groups, stating that the chitosan and gelatin scaffold has slower degradation rate when compared with PerioCol.

The biodegradability of the chitosan and gelatin-based scaffold was attributed to the presence of glycosidic bonds in their molecular structures that can be broken by enzymatic action. Lysozyme breaks the glycosidic bonds in chitosan and gelatin leading to the degradation of chitosan and dissolution of gelatin into smaller fragments, leading to the formation of non-toxic oligosaccharides and amino acids that are biocompatible and non-toxic in nature [20]. The oligosaccharides and amino acids produced during chitosan and gelatin degradation can have variable lengths and depending on their size and chemical structure, they may either be incorporated into metabolic pathways within the body or excreted from the body through normal waste elimination processes [21]. These aspects make these scaffolds valuable materials in applications where controlled biodegradability is desired.

The surface characteristics and adsorption potential of chitosan and gelatin-based scaffolds immersed in different body fluids were assessed using SEM analysis [22]. The chitosan and gelatin-based scaffold immersed in saliva showed a translucent suspension where the surface of the scaffolds appeared smooth and flat without any noticeable cracks and irregularities, which suggests that the scaffold materials interacted with saliva, resulting in a visible change in the appearance of the surface. The scaffold immersed in anticoagulant blood revealed some irregularities on the scaffold surface and these irregularities were attributed to the adsorption of hematologic proteins, such as albumins and globulins, onto the scaffold surface and suggested that the scaffold has a tendency to bind or adsorb these proteins from the blood. The irregularities are explained by the chelation of coagulants in the blood with calcium present in the anticoagulant. This interaction between calcium ions and coagulants may result in surface changes on the scaffold [23]. The scaffold present in the blood without any added anticoagulant shows larger clumps of dried blood on the surface of the scaffold, resulting in a little irregular surface of the scaffold. The interaction of blood with the outer surface of the scaffold resulted in a rough and irregular surface of the scaffold with an absence of pores as the coagulation of blood had resulted in the closure of pores on the scaffold's surface. The SEM observations state that the chitosan and gelatin-based scaffold has good adsorption in various body fluids attributing to the superior biocompatibility of the scaffold. 

Chitosan and gelatin are combined with various materials which enhances the strength and mechanical properties of the scaffold. The chitosan and gelatin-based membrane has a porous nature and greater biodegradability when compared with other commercially available membranes [24]. The superior biodegradability and porosity allow the absorption of various body fluids, making the scaffold suitable for use as a barrier membrane in regenerative procedures where controlled fluid absorption and tissue regeneration are needed.

Limitations of the study

This was an in vitro study where only two properties of the scaffold were assessed. Further in vivo and randomized controlled trials should be done on the scaffold for its future applications. 

## Conclusions

The present study confirms that the chitosan and gelatin-based scaffolds have slower biodegradability when compared with the available GTR membrane named PerioCol. The scaffold remains for a longer period of time and can be used as a local drug delivery agent for delivering therapeutic agents to the surgical site. Because of the pore size and regulation in biodegradability, this scaffold enhances periodontal regeneration by allowing selective repopulation in the periodontal pocket.
